# Profiles of volatile indole emitted by *Escherichia coli* based on CDI-MS

**DOI:** 10.1038/s41598-019-49436-y

**Published:** 2019-09-11

**Authors:** Qiaoshi Zhong, Feng Cheng, Juchao Liang, Xiaozhong Wang, Yanhui Chen, Xueyao Fang, Longhua Hu, Yaping Hang

**Affiliations:** 1 Department of clinical laboratory, Jiangxi Province Key Laboratory of Laboratory Medicine, The Second Affiliated Hospital of Nanchang University, Nanchang, 330006 P.R. China; 2 Department of clinical laboratory, Jiangxi Chest (third people) Hospital, Nanchang, 330006 P.R. China; 3 Jiangxi Key Laboratory for Mass Spectrometry and Instrumentation, East China Institute of Technology, Nanchang, 330013 P.R. China

**Keywords:** Diagnostic markers, Bacterial infection

## Abstract

*Escherichia coli* is an important pathogen of nosocomial infection in clinical research, Thus, exploring new methods for the rapid detection of this pathogen is urgent. We reported the early release of molecular volatile indole vapour of *E. coli* cultures and blood cultures analyzed by direct atmospheric corona discharge ionization mass spectrometry (CDI-MS). The concentration of indole in *E. coli* cultures remarkably increases during the early log and lag phases of bacterial growth, thereby enabling early detection. Technical replicates were cultivated for 3 days for reference diagnosis using current conventional bacteraemia detection. A reference MS screen of common microbes from other genera confirmed that the peaks at *m/z* 116 signal corresponded to indole were specifically present in *E. coli*. Our results indicated that volatile indole based on CDI-MS without the need for any sample pretreatment is highly suitable for the reliable and cost-efficient differentiation of *E. coli*, especially for bacteraemia in humans.

## Introduction

*Escherichia coli* (*E. coli*) is an opportunistic pathogen that is frequently isolated from urinary tracts (46.4%), surgical burns and wounds (20.6%), blood stream (22.2%), other sterile body fluids (21.0%) and pulmonary tracts (5.0%), according to the 2017 CHINET monitoring network. Furthermore, *E. coli* infections are one of the most significant causes of death in the early neonatal period^1^. In China, the isolation rate of hospital-acquired *E. coli* infections ranked first with an average of over 19% for 12 years^2^. *E. coli* has developed extremely high resistance to common antibiotics, and targeted medical approaches are required to efficiently treat its associated diseases. However, such therapies increase the probability of spreading drug-resistant infections^3^. Therefore, the accurate and rapid detection or monitoring of this pathogen is a critical step for the adequate treatment of infection.

Conventional methods, such as selective medium screening and biochemical tests, remain the gold standard in detecting bacteria. However, serological tests, PCR and loop-mediated isothermal amplification suffer the drawback of needing to be coupled to culture enrichment procedures to ensure the detection of metabolically active cells^4,5^. These steps are often laborious and slow. Several sophisticated mass spectrometry (MS) technologies have emerged and can be used to recognize volatile organic compounds (VOCs). GC-MS is an appropriate and reliable technique for the isolation and identification of VOCs^6^. Secondary electrospray ionization-MS (SESI-MS), with the ability to directly sample gas, exhibits some of the best features of a desired laboratory analytical tool^7^. MALDI-TOF MS has been widely accepted and gradually applied in clinical laboratories and is based on the differences of bacterial protein spectra^8,9^. These three different methods exhibit their own shortcomings^10^. In our study, the evident potential advantages of homemade and new high-sensitivity ambient corona discharge ionization MS (CDI-MS) is ultimately rapid (several hours), accurate and with high-throughput without any pretreatment (ca. 10–20 s per sample). Therefore, the method is suitable for simple real-time analysis and the potential for direct bacteria screening^10,11^. Furthermore, this method could simultaneously detect both polar and non-polar analytes with high sensitivity, showing promising potential as an application for the rapid detection of various compounds present in complex matrices^12^.

VOCs generate characteristic odours for certain bacteria, and these VOCs can be used for species identification and non-inasive medical diagnostics^6,13–15^. As shown in Table 1, several *E. coli* metabolites previously observed by MS showed potential advantages. Indole is an organic compound that is widespread in microbial communities inhabiting diverse habitats, such as the soil environment and human intestines^16^. Indole is synthesized by tryptophanase, the enzyme that catalyzes L-tryptophan conversion to indole, and is encoded by the *tnaA* gene, a mutant of *E. coli* that lacks *tnaA* and cannot produce its own indole^17^. Measurement of indole production is a traditional method for the identification of microbial species. Our studies strongly indicate the excellent potential of indole detection for the rapid recognition of *E. coli* compared with common clinical diagnostic routines (6–7 h vs. 2–3 days). Indole detection is realized using the widely available type of CDI-MS instrumentation with cost efficiency, speed and high simplicity. This study identifies the common and unique volatile biomarkers for *E. coli* investigation by using non-separative CDI-MS.

**Table 1:** *E. coli* VOCs previously observed by mass spectrometry.

Biomarker	Method	Specificity	Time (*h*)
Indole	GC-MS^22^	*E. coli* O157:H7	16–72
Acetonitrile/Ethanol/Indole	SESI-MS^23^	*E. coli*/*S. aureus*/*P. aeruginosa*	24
Ethanol/1-Propanol/Indole	GC-MS^24^	*E. coli*/*S. Typhimurium*	4–10(shake)
Acetaldehyde/2,3-Butadione/Ethyl acetate	GC-MS^25^	*E. coli*/*S. aureus*	14
*m/z* 65, 91, 92, 117, 118,119	SESI-MS^7^	*E. coli*/*S. aureus*/*S. Typhimurium*	8
1-Decanol/Indole	HS-SPME-GC-MS^26^	*E. coli*/*K. pneumoniae/S. aureus*	19
1,9-Decadiene, 2-Acetyl-1-pyrroline,/2-Heptanone/2-Methyl tetradecane/Indole	SPME GC-MS^13^	*E. coli*/*S. aureus*/*Candida albicans*	24
Indole	HS-SPME-GC-MS^27^	*E. coli* O157:H7	12

## Results and Discussion

### High-resolution CDI-MS and CID analysis of *E. coli* volatiles

Our previous study has introduced almost all common clinical bacterial VOCs *in vitro* by ambient MS^18^, However, no conclusive structural attribute for m/z 116 was made in our preliminary work. *E. coli* colonies smell especially sour. Considering the prevalence and drug resistance of *E. coli* in bloodstream infection, we focused on researching the VOCs released by clinical *E. coli*. A total of 5 cases among 35 suspects were identified as *E. coli*, which were consistent with the results of traditional biochemical analysis that confirmed the potential of ambient MS for clinical practice. Based on our previous studies and current experiments, no characteristic signal of VOCs released by *E. coli* showed in the positive mode.

**Figure 1:**
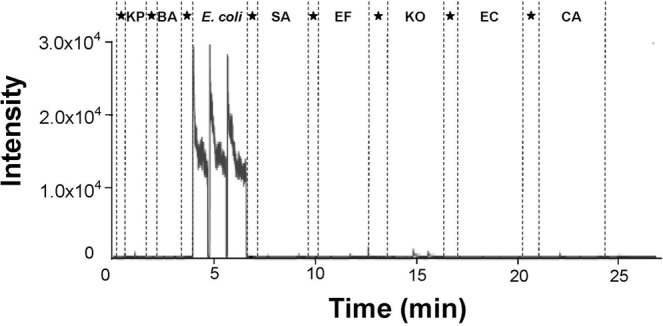
Single ion chromatogram for *m/z* 116 signals in the CDI-screen of *E. coli*. 1 strain were analyzed three times in succession. The samplings of other strains were *Klebsiella pneumonia*(KP), *Acinetobacter baumannii*(BA), *Staphylococcus aureus*(SA), *Enterococcus faecalis*(EF)*, Klebsiella oxytoca*(KO), *Enterobacter cloacae*(EC), and *Candida*(CA); Asterisk(★): blank background reference (A centrifuge tube containing MH without *E. coli* cultured over the same time period was used as background spectra).

**Figure 2:**
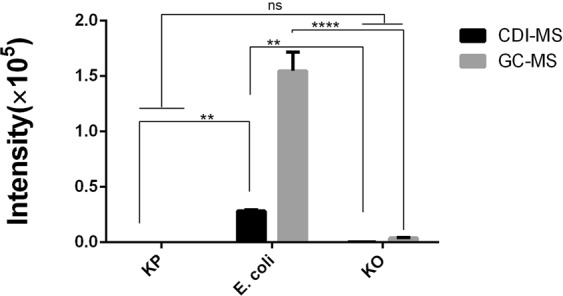
CDI-MS and GC-MS measurement of indole signals obtained from the VOCs emitted by KP (indole non-producing), *E. coli* and KO(indole-producing). Two-way ANOVA was performed to calculate statistical significance. Values that are significantly different are indicated by asterisks as follows: ns, no significant **P < 0.01; ****P < 0.0001. *n* = 3 per group. Error bars indicate standard deviations (SD).

The VOC peaks of *E. coli* were screened by positively diagnosed specimens. The baseline level of ion chromatograms corresponds to the purify centrifuge tube. After subtracting corresponding signal of the blank background reference (pure centrifuge tube), the spectrum intensity at *m/z* 116.17 observed was greatly increased in the headspace of *E. coli* cultures due to the volatility of the ionized chemicals (10 cultures/11 blood cultures). The intensity added up to the 10^4^ level (Fig. 1). The reference CDI-MS screening of common infectious microbes, including *Klebsiella pneumonia*(KP), *Acinetobacter baumannii*(AB), *Staphylococcus aureus*(SA), *Enterococcus faecalis*(EF)*, Klebsiella oxytoca*(KO), *Enterobacter cloacae*(EC), and *Candida*(CA), did not show notable release of indole (Fig. 1)^18^. VOCs of KP (indole non-producing), *E. coli* and KO (indole-producing) were tested by GC-MS and CDI-MS, respectively. The results showed that indole signal of KP was not detected by both methods and could not be distinguished from KO. While, through the GC-MS method of complex SPME extraction and enrichment showed that the indole signal intensity of KO were still low, but the indole signal of *E. coli* were greatly enhanced, suggesting that the efficacy of indole production by *E. coli* is much higher than other bacteria. In addition, both methods can better distinguish *E. coli* by indole from other indole non-producing bacteria (Fig. 2). These results indicated the high specificity of indole to *E. coli* by CDI-MS.

**Figure 3:**
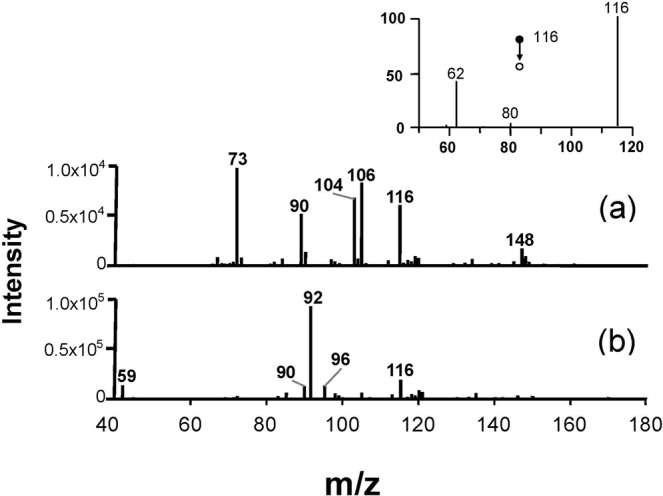
CDI-MS spectrum of VOCs emitted by *E. coli* culture after 16 h incubation in negative ionization mode. (**a**) CID-MS spectrum of *m/z* 116; (**b**) VOCs spectrum of microbial culture; (**c**) VOCs spectrum of blood culture.

Based on high-resolution measurements (Orbitrap XL, Thermo Scientific, San Jose, CA, USA), the chemical composition of *m/z* 116 ion was determined to be C8H6N^−^, which is the metabolite formula that best fits this value within the mass accuracy of detection. Structurally, *m/z* 116 ion was identified as deprotonated indole [M-H]^−^ based on the exact match of CID patterns for the *m/z* 116 signal produced by *E. coli* cultures (inset in Fig. 3a). The structural CID analysis of C8H6N^−^ ion displayed major characteristic fragments at *m/z* 80 and 62 (Fig. 3a). Under the same experimental conditions, we performed reference analysis of model VOCs discovered in bacteria, humans and other living organisms that can form C8H6N^−^ ions upon deprotonation^19,20^. Organic acids, such as acetic, butyric and isovaleric acids, were preferentially ionized from the headspace of blood cultures via proton abstraction^18^. In view of the results of CID experiments and other research, This finding strongly indicates that the C8H6N^−^ ion observed in the volatile headspace of *E. coli* cultures was formed by the deprotonation of indole.

A number of signals, that is, *m/z* 73, 104, 106, 148 (Fig. 3b); and *m/z* 59, 92, and 96 (Fig. 3c), are shared among different bacteria. The peaks detected in background-subtracted spectra do not necessarily belong to bacterial VOCs. Some peaks in the spectra belong to blood or MH metabolites released in response to inoculation. Apart from the signals mentioned above, the *m/z* 90 detected at high intensity in CDI-MS of *E. coli* corresponded to radical water cations formed via electron abstraction rather than protonation. Thus, ambient ionization by corona discharge can be considered as a highly selective and sensitive approach to detect indole in *E. coli* cultures.

### Ionization mechanism of indole

The prevalence of indole over the rest of VOCs signal in *E. coli* is remarkable. The ionization of bacterial volatiles by CDI in our experiments most likely occurs via a similar mechanism to that in atmospheric pressure chemical ionization^18^. It is well known that ambient corona discharge possesses a much higher ionization efficiency than ESI in the case of analyzing small molecules, especially for detecting compounds of low proton affinity and/or low/no polarity^12^. The signal formation was heavily dependent on the proton affinity (PA) and the ionization energy (IE) values of the analytes. Indole exhibits a low or medium-level polarity and high vapour pressure. Note that indole has high PA (933.4 kJ/mol), but it gives radical cations rather than the protonated molecules in this study, probably because indole has a low IE (7.76 eV) and no suitable site to accept the proton in our experimental conditions. Moreover, The ion production is largely related to the unique process of ambient corona discharge, which probably produces energetic species such as water radical cations, nitrogen radical cation, etc^12^.

### Time profile of indole release by *E. coli*

**Figure 4:**
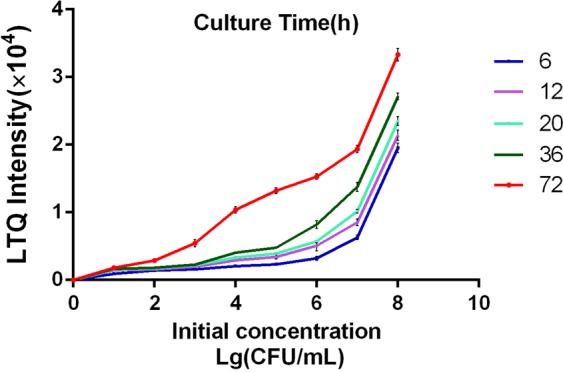
Time profiles of indole signal (*m/z* 116) detected by CDI-MS from the headspace of simulated blood cultures with different initial concentrations of *E. coli* (negative ionization mode). *n* = 3 per group. Error bars indicate standard deviations (SD).

The release of indole by *E. coli* cultures followed a highly characteristic transient time profile. Figure 4 shows the time profiles of indole signal from the headspace of cultures with different initial counts of *E. coli* (10^1^–10^8^ CFU/mL). Each time point for different initial *E. coli* concentrations was analyzed by CDI-MS using at least four independently grown cultures. In all the microbe cultures, indole production was substantially enhanced during the early culture time of bacterial growth (6–7 h, Fig. 4). We also detected that the early onset of indole release depended on the high initial counts of *E. coli* (Fig. 4). The initial increase of indole level in *E. coli* cultures was followed by a steady increase lasting for >36 h and 10^5^ CFU/mL then a sharp increase. Indole is produced by various bacterial species, including *E. coli* and *Vibrio cholerae*^16^. *E. coli* can produce millimolar concentrations of indole from tryptophan by means of tryptophanase (TnaA) during the stationary growth phase under nutrient-rich conditions^16^. The ability of indole to be excreted into the extracellular medium is strong and indole has good volatility, thus the growth rate of *E. coli* can be selectively enhanced by gentle agitation to increase the signal response of indole for targeted analysis, regulated by numerous pathways that typically connect growth to nutrient availability (free tryptophan)^21^. Thus, the enhancement of indole emission during the early culture time of bacteria is particularly beneficial for the rapid identification of *E. coli*.

### Outlook

The signal of indole in blood culture was weaker than that in solid culture (Fig. 3). This weak signal accounts for high antibacterial blood immunity and low bacteria resistance. Thus, low inter-individual signal variability and medium selection are crucial for the quantitative measurement of *E. coli* concentration in culture solution. The detection time in our study is greatly shorter than that in traditional routines. Further analytical improvement can also be performed. Matrix effects, most importantly those associated with solution pH, change in culture environment or optimization of MS conditions, must also be considered. Therefore, the calibration curve and cut-off value for quantitative indole testing as the clinical sample must be established. The standard method is recommended for the reliable and accurate quantification of indole in patient cultures based on the CDI-MS analysis of volatiles. Moreover, this technique has research potential for detection complex matrices. The detailed application required further studies to directly analyze complex samples such as urine or sputum without matrix separation.

## Conclusion

In this study, we used tandem analysis and confirmed that the *m/z* 116 biomarker signal in the cultures of *E. coli* corresponded to indole. The continuous improvement of the property of non-invasive CDI–MS instruments and the development of highly targeted approaches are superior over the time-consuming traditional clinical routines for alternative analysis.

## Materials and Methods

### Ethical approval and informed consent

All experiments were performed in accordance with the guideline “Biomedical research ethics review method involving people” of China and were approved by the medical research ethics committee at the Second Affiliated Hospital of Nanchang University. Informed consents were obtained from the participants.

### Conventional identification and preservation of pathogens

We observed ten clinical *E. coli* isolates, Eleven blood cultures had been identified as *E. coli* and thirty-five suspected bacteraemia blood cultures were collected from patients in the Second Affiliated Hospital of NanChang University. The specimens had been previously screened for the presence of *E. coli* by VITEK2-compact (BioMerieux). The samples were preserved in frozen milk at −80 °C.

### Sample preparation

#### Headspace volatiles of bacteria culture

Approximately 6–8 monocultures of preserved strains on blood agar plates were picked and transferred to MH centrifuge tubes (Solarbio, Beijing) or SPME vial (ANPEL, Shanghai). A centrifuge tube/SPME vial containing MH without bacteria cultured over the same time period was used as background spectra.

#### Headspace volatiles of bacteria blood culture

Model blood culture: negative blood cultures for reference were selected based on the lack of bacteraemia signs. Each negative blood culture was collected completely (48 mL) in Hemoline (BioMerieux), and 20 mL liquid was removed and equally divided into four fractions. Three out of the four fraction was inoculated with 1 mL of *E. coli* suspension diluted by 0.45% sterile NaCl solution. *E. coli* simulated blood cultures were grown at different incubation time (6–10 time points for a total of 72 h) and different original concentrations (10^1^–10^8^ CFU/mL). The remaining fraction was replaced with 1 mL of 0.45% sterile NaCl solution. All the fractions were incubated in the 10 mL centrifuge tubes (Solarbio). Suspected bacteraemia blood culture was incubated under the same condition for 0–16 h. Another 1 mL was used for biochemical identification. Approximately 5 mL of suspected and simulated blood cultures, and *E. coli* blood cultures were directly detected by MS analysis. After different times and original concentrations, the time growth curves of VOCs were observed.

### MS analysis of bacteria VOCs

#### Detecting by GC-MS

1. Extraction of volatile substances
  The VOCs were enriched by headspace solid phase microextraction (SPME). The 50/30 nm DVB/CAR/PDMS extraction head was aged at 270 °C for 30 min at the inlet of the GC-MS. The aging SPME extraction head was inserted into the headspace vial, and the sample was equilibrated in a 40 °C water bath for 30 min, and then the fiber head was pushed out so that the fiber head was in the upper position of the top of the headspace vial, The 40 °C water bath is adsorbed for 30 min, the fiber head is taken back from the extraction head, and the headspace vial is pulled out and transferred to the GC-MS inlet.
2. GC-MS
  The SPME extraction head was injected into a Agilent 7890B-5977A GC containing a 30 m × 0.25 mm × 0.25 μm HP-5MS column (Agilent), and the fiber head was analyzed at 250 °C for 2 min.

GC setting: starting temperature is 33 °C, hold for 3 min, heat up to 180 °C at 10 °C/min speed, heat up to 240 °C at 40 °C/min speed, keep 4 min, carrier gas is Helium. The running time is 23.2 min. MS setting: the ion source temperature is 230 °C, the ionization mode is EI source, the energy is 70 eV, and the whole scanning mode is used for detection. The scan mass range was from m/z 50 to 400, and the scan time was 0.1 s. The VOCs repeatedly detected in the sample headspace vial, and subtracted the substance detected in the background control, so as to the VOCs produced by the bacteria. Mass spectra of VOCs were compared to those obtained from the NIST14s library.

#### Detecting by CDI-MS

The discharge stainless steel needle was maintained in high voltage (+4 kV in positive and −3.5 kV in negative ion detection modes, respectively) to create corona at ambient pressure. For CDI-MS analysis, the open centrifuge tube with microbial or blood culture was connected to the mounted cap. Changing the sample simply required disconnecting the tube and jointing a tube with the new sample to the same cap. This operational workflow permited high reproducibility and throughput of analysis. The headspace VOCs of cultures were continuously diverted into the ionization region with nitrogen gas flow (0.1 Mpa, 1 L/min). The device from the inlet of the culture to the inlet of the LTQ (ThermoFischer) capillary was previously introduced in detail^10^.

LTQ-Tune automatically optimized detection systems, the ion lens, and other parameters. Three replicate samples were independently prepared and analyzed for incubation. CDI-MS analysis of each replicate was acquired for 30–60 s in the *m/z* range of 15–200. Each fingerprint is averaged from the 120 scans acquired over the initial sampling period of 10 s. VOCs of reference standard compounds were analyzed from aqueous solutions with identical experimental conditions. Collision-induced dissociation (CID) analysis of indole and reference compounds were detected on LTQ-XL MS (Thermo Scientific) with a collision energy (CE) of 10–30%, a mass-to-charge window width of 1.5 units and on the characteristic ions for molecular structure identification.
